# Treatment in Leucorrhœa in Little Girls

**Published:** 1880-09

**Authors:** 


TREATMENT OF LEUCORRHŒA IN LITTLE GIRLS. (*Bouchut, Paris Méd and Le Pratician*, No. 17, p. 202.)

The author denies that the leucorrhœa is produced by vaginitis or metritis, but is always the result of vulvitis, and it is the latter that should be treated. He therefore rejects vaginal injections which are difficult to make in little girls, as well as repugnant to the mothers, and simply treats the vulva.

The treatment is both local and general.

*Locally.*—Extreme cleanliness of the diseased parts, obtained by means of repeated lotions with bran water of walnut leaves, Goulard's water, etc. 2nd, *modify the diseased surfaces.* For this purpose the means are numerous, corrosive sublimate (10 centigr. to 300) in sitz baths or lotions; carbolic acid, 5 to 1000; coal tar diluted one-half and finally cauterization with a solution of nitrate of silver (20 centigr. to 30 grams) are useful. Between the lotions it is well to apply a pledget of lint saturated with coal tar or an ointment of red precipitate between the labia. This, kept in place by a pad, protects the labia and surrounding parts from irritation from the pus which is often very abundant.

Internally the author recommends for strumous patients cod-liver oil and quinine, and for herpetic patients arsenic. The disease is often rebellious to treatment.

